# Hybrid Transverse Polar Navigation for High-Precision and Long-Term INSs

**DOI:** 10.3390/s18051538

**Published:** 2018-05-12

**Authors:** Ruonan Wu, Qiuping Wu, Fengtian Han, Rong Zhang, Peida Hu, Haixia Li

**Affiliations:** Department of Precision Instrument, Tsinghua University, Beijing 100084, China; wrn13@mails.tsinghua.edu.cn (R.W.); rongzh@mail.tsinghua.edu.cn (R.Z.); hpd07@mails.tsinghua.edu.cn (P.H.); li-hx03@mails.tsinghua.edu.cn (H.L.)

**Keywords:** polar navigation, transverse frame, long-term error propagation, high-precision INSs, Earth-fixed frame

## Abstract

Transverse navigation has been proposed to help inertial navigation systems (INSs) fill the gap of polar navigation ability. However, as the transverse system does not have the ability of navigate globally, a complicated switch between the transverse and the traditional algorithms is necessary when the system moves across the polar circles. To maintain the inner continuity and consistency of the core algorithm, a hybrid transverse polar navigation is proposed in this research based on a combination of Earth-fixed-frame mechanization and transverse-frame outputs. Furthermore, a thorough analysis of kinematic error characteristics, proper damping technology and corresponding long-term contributions of main error sources is conducted for the high-precision INSs. According to the analytical expressions of the long-term navigation errors in polar areas, the 24-h period symmetrical oscillation with a slowly divergent amplitude dominates the transverse horizontal position errors, and the first-order drift dominates the transverse azimuth error, which results from the $g^{0}$ gyro drift coefficients that occur in corresponding directions. Simulations are conducted to validate the theoretical analysis and the deduced analytical expressions. The results show that the proposed hybrid transverse navigation can ensure the same accuracy and oscillation characteristics in polar areas as the traditional algorithm in low and mid latitude regions.

## 1. Introduction

Nowadays, the increased exploration of polar areas makes the high-precision navigation of airplanes and vessels at high latitudes an urgent requirement in economic, military and research fields [1,2]. Although there are abundant navigation methods at low and mid attitudes, most of them (e.g., radio navigation, satellite navigation, magnetic compasses and celestial navigation) are limited, or even invalid, in polar regions due to the special geometry and magnetic field required [3,4,5,6,7]. Fortunately, inertial navigation is based on dead reckoning with almost no need for external information [8], which makes inertial navigation systems (INSs) insensitive to the outside environment. Thus, INSs should be able to stably provide high-precision navigation solutions at high latitudes. However, some problems have to be solved first [9,10]. Firstly, the convergence of meridians causes a significant increase in longitude error and azimuth error at high latitudes and even the invalidity of the heading reference near the poles. Secondly, the rates of velocity and azimuth are predicted to sharply increase when the system passes through the poles, which could cause overflow during computing.

The primary cause of such problems is that the widely used definition of the navigation frame, namely, the geographic frame (*n*-frame), relies on the geographic distribution of parallels, meridians and the poles. A representative solution is to choose other navigation frames of which the definitions are independent of such geographic distribution. The grid system [11] and transverse system [12] have been proposed to achieve this goal. These two systems are based on a grid frame and a transverse frame, respectively, which have become widely used in recent research related to polar navigation. Although the practical differences between the two systems are inconspicuous, the transverse system is easier to visualize and less complicated [12]. Its basic idea is to move the poles to somewhere on the equator. Thus, the new equator will run through the traditional poles, and the advantages of equatorial navigation could be obtained in polar navigation [12]. However, as the shape of the Earth is closer to an ellipsoid than to a sphere, the standard transverse system is limited by the principle error resulting from the spherical assumption. Yao et al. [13] and Li et al. [14] modified the definition of the transverse frame under the ellipsoidal Earth model. These two works used different normals to indicate the local-level plane, and they also have different definitions of transverse latitude and transverse longitude. The modified transverse frame shown in reference [13] has similar advantages to the *n*-frame—able to indicate the movement and attitude relative to the surface of the Earth and to cooperate with external auxiliary information.

It should be noted that neither the grid system nor the transverse system has the ability to navigate globally. When the vehicle locates near the intersection points of the 90° W and 90° E meridians with the equator, both of the two systems face similar problems to the *n*-frame system in polar areas. As the *n*-frame is widely used at low and mid latitudes, the system needs a complicated switch between the polar navigation algorithm and the traditional algorithm when it moves across the polar circles, which would introduce a sudden change in the core integral process. Zhou et al. [15] and Yao et al. [16] have proposed indirect polar navigation methods, using a combination of the wander navigation system and grid navigation or the transverse navigation to achieve inner consistency. However, the wander attitude reference can be employed only in some polar regions because it would become invalid at latitudes of ±90° [17]. Such a problem can be solved by choosing a navigation frame that is completely independent of the shape of the Earth and the system’s position. Based on this thought, Liu et al. [18] employed the pseudo INS mechanization to constitute a global navigation algorithm. The navigation calculation unit is executed in the pseudo-Earth frame under the spherical Earth model, which matches the standard transverse frame but is not a commonly-used mechanization in general INSs. In addition, the navigation calculation outputs under the spherical Earth model need to be corrected and transformed to the ellipsoidal Earth model for human–machine interaction and communication [18]. In order to achieve the same or better performance of global navigation with the least change in the existing systems, we would like to improve the algorithm one step further using the simpler, and more frequently used, Earth-fixed mechanization directly under the ellipsoidal Earth model.

In order to improve the precision of polar navigation, more factors have been considered. For example, an accurate initial alignment is essential for INSs. Gao et al. employed the unscented Kalman filter to accomplish the polar alignment based on the pseudo-Earth frame [19]. Cheng et al. used the adaptive unscented Kalman filter with the aid of a star sensor to improve the accuracy of polar transfer alignment for conditions where the master INS is inaccurate and the information from the system model is abnormal [20]. Wang et al. proposed a polar transfer alignment algorithm based on an improved adaptive Kalman filter, which aimed to solve the problem caused by lever-arm and flexural deformation [21]. Moreover, Yan et al. established an integrated navigation system to improve the polar navigation accuracy of unmanned underwater vehicles [22]. As for the high-precision and long-term INSs, it is not enough to merely provide the pure-inertial mechanization and error models. The analysis of damping technology and error propagation is also essential. Li et al. [23] proposed a polar damping technology for the transverse strapdown INS based on the system’s static error characteristics. Huang et al. designed a damping algorithm based on the Kalman filter to reduce the overshoot errors due to the switch from the non-damping state to the damping state [24]. In this paper, the damping technology is discussed, and two more aspects are taken into consideration: First, outside of the polar areas, the kinematic error characteristics could be analyzed using static models, as the system’s angular velocity around the Earth’s axis is far less than the Earth’s rotational rate. However, in polar areas, the movement around the Earth’s axis is non-ignorable compared with the Earth’s rotation and thus, results in a change in the error characteristics, which has not been addressed in existing works. Second, a damping method that does not increase the complexity of the navigation algorithm switch is preferred. In addition, navigation errors of INSs are induced by multiple error sources. Finding out long-term contributions of main error sources is essential for the design of high-precision INSs [25], which is barely discussed in the polar navigation themed existing works and will be addressed in this paper.

This research focuses on a hybrid polar navigation method which has a more concise form and will be easier to implement. A comprehensive analysis of this method is proposed including the basic mechanism, the pure-inertial error model, the discussion of proper damping technology and the corresponding long-term error propagation for high-precision INSs. The rest of this paper is arranged as follows: In Section 2, the oscillation of traditional horizontal channels in polar areas is reanalyzed considering the longitude rate. Then, the pure-inertial hybrid transverse polar navigation method and its error models are proposed in Section 3 and Section 4, respectively. In Section 5, the damping technology is discussed based on the kinematic error characteristics. The analytical expressions of the long-term navigation errors are deduced in Section 6. The simulation results are discussed in Section 7. Finally, conclusions are drawn in Section 8.

## 2. Traditional Horizontal Oscillation in Polar Regions

Before the polar navigation method for INSs is discussed, it should be noted that the convergence of meridians will cause other problems besides the rapid growth of errors. The system’s longitude rate ($\dot{\lambda }$) is a function of the eastern velocity ($v_{E}$) and latitude (*L*) [26]:

(1) $$\dot{\lambda }=\frac{v_{E}}{\left(R_{N}+h\right)\cos L},$$

where *h* is the height above the reference ellipsoid and $R_{N}$ is the normal radius of curvature taken in the direction of the prime vertical, given in [26,27]

(2) $$R_{N}=\frac{a}{{\left(1-e^{2}\sin^{2}L\right)}^{1/2}},$$

where *a* is the semi-major axis and *e* is the first eccentricity of the reference ellipsoid.

In Equation (1), cos *L* decreases along with the increase of latitude and will tend towards zero as the latitude approaches ±90°. At low or mid latitudes, $\dot{\lambda }$ is far less than the Earth’s rotational rate ($\omega_{ie}$). Therefore, the low speed kinematic error characteristics can be represented using static error models. However, when the system moves along a certain parallel near the poles, the considerable magnification of $\dot{\lambda }$ leads to a difference between the static and kinematic oscillation characteristics in the system’s horizontal channels.

Based on the *n*-frame error models of the pure-inertial navigation [8,26,28], variables involving $\dot{\lambda }$ remain, while other relatively small quantities and the coupling between the vertical and the horizontal channels are neglected. Thus, the state equation of the kinematic errors in horizontal channels is simplified as

(3) $$\left(\begin{array}{c} \delta {\dot{r}}_{N}\\ \delta {\dot{r}}_{E}\\ \delta {\dot{v}}_{N}\\ \delta {\dot{v}}_{E}\\ {\dot{\psi }}_{N}\\ {\dot{\psi }}_{E}\\ {\dot{\psi }}_{D} \end{array}\right)=\left(\begin{array}{ccccccc} 0 & -\dot{\lambda }\sin L & 1 & 0 & 0 & 0 & 0\\ \dot{\lambda }\sin L & 0 & 0 & 1 & 0 & 0 & 0\\ -\omega_{s}^{2} & 0 & 0 & -\omega_{1} & 0 & g & 0\\ 0 & -\omega_{s}^{2} & \omega_{1} & 0 & -g & 0 & 0\\ 0 & 0 & 0 & 0 & 0 & -\omega_{2} & 0\\ 0 & 0 & 0 & 0 & \omega_{2} & 0 & \omega_{3}\\ 0 & 0 & 0 & 0 & 0 & -\omega_{3} & 0 \end{array}\right)\left(\begin{array}{c} \delta r_{N}\\ \delta r_{E}\\ \delta v_{N}\\ \delta v_{E}\\ \psi_{N}\\ \psi_{E}\\ \psi_{D} \end{array}\right)+\left(\begin{array}{c} 0\\ 0\\ \nabla_{N}\\ \nabla_{E}\\ \varepsilon_{N}\\ \varepsilon_{E}\\ \varepsilon_{D} \end{array}\right),$$

where

(4) $$\begin{array}{l} \omega_{1}=\left(2\omega_{ie}+\dot{\lambda }\right)\sin L\\ \omega_{2}=\left(\omega_{ie}+\dot{\lambda }\right)\sin L\\ \omega_{3}=\left(\omega_{ie}+\dot{\lambda }\right)\cos L \end{array}\}.$$

In Equation (3), ${\delta r}_{N}$ and ${\delta r}_{E}$ are the Northern and Eastern position errors, ${\delta v}_{N}$ and ${\delta v}_{E}$ are the Northern and Eastern velocity errors, $\psi_{N}$, $\psi_{E}$ and $\psi_{D}$ represent the Northern, Eastern and vertical components of the psi angle (the psi angle is a rotation vector that reflects the orientation difference between the computed version and the actual version of the navigation frame due to gyro errors [29]), $\omega_{s}$ is the Schuler frequency, $\nabla_{N}$ and $\nabla_{E}$ are the Northern and Eastern accelerometer errors, $\varepsilon_{N}$, $\varepsilon_{E}$ and $\varepsilon_{D}$ are the Northern, Eastern and vertical components of gyro drift errors, and *g* is the gravity acceleration.

Using $A_{7\times 7}$ to denote the coefficient matrix in Equation (3), the characteristic equation can be calculated from $\left|\mathrm{sI}-A_{7\times 7}\right|$ (I denotes the 7×7 unit matrix), as

(5) $$s\left(s^{2}+{\tilde{\omega }}^{2}\right)\left[s^{4}+2\left(\Omega^{2}+2{\tilde{\omega }}^{2}\sin^{2}L\right)+\Omega^{4}\right]=0,$$

where

(6) $$\begin{array}{l} \Omega^{2}=\omega_{s}^{2}-\dot{\lambda }\sin^{2}L\left(\dot{\lambda }+2\omega_{ie}\right)\\ \tilde{\omega }=\omega_{ie}+\dot{\lambda } \end{array}\}.$$

The roots of the characteristic equation consist of zero, a pair of purely imaginary numbers ± *j*($\dot{\lambda }$ + *ω_ie_*) and two pairs of complex numbers whose angular frequencies are approximately *ω_s_* ± ($\dot{\lambda }$ + *ω_ie_*)sin *L*. In contrast to the conditions at low and mid latitudes, the distribution of characteristic roots in polar areas is also influenced by the longitude rate, $\dot{\lambda }$. Thus, the horizontal channels would have two kinds of oscillation characteristics according to the magnitude of $\dot{\lambda }$.

(1) When the system situates in polar regions but is still away from the poles, the order of magnitude of $\dot{\lambda }$ is similar to $\omega_{ie}$ and much smaller than $\omega_{s}$ (e.g., $\dot{\lambda }$ = 8.95 × 10^−5^ rad/s for $v_{E}=5\;m/s$ at 89.5° N). In the short term, $\left(\dot{\lambda }+\omega_{ie}\right)\sin\;L$ can be neglected and the oscillations in horizontal channels are mainly Schuler oscillations. Therefore, the traditional damping network is still workable, and the angular frequency of the damped horizontal channels is $\dot{\lambda }$ + *ω_ie_*, which means more drastic fluctuation in error curves.

(2) If the system is close enough to the poles, the order of magnitude of $\dot{\lambda }$ is similar to, or even bigger than, $\omega_{s}$ (e.g., $\dot{\lambda }$ = 0.045 rad/s for $v_{E}=5\;m/s$ at 89.999° N). Then, the angular frequencies of the complex roots, namely $\dot{\lambda }$ + *ω_ie_* and $\omega_{s}\pm \left(\dot{\lambda }+\omega_{ie}\right)\sin\;L$, are mainly determined by $\dot{\lambda }$. This means that the fluctuation in horizontal channels will be far more drastic than Schuler oscillations. Therefore, the traditional damping network is invalid.

In summary, the problems that traditional INSs are confronted with in polar areas include not only the extremely large longitude error and azimuth error, but also more drastic fluctuation in the horizontal channels and even the invalidity of existing damp techniques due to the system’s movement. A polar inertial navigation algorithm should solve both the problems.

## 3. Mechanism of Hybrid Transverse Navigation

Fundamentally, all the problems that the traditional inertial navigation algorithm encounters in polar areas result from the convergence of meridians. If definitions of all the coordinates used in the navigation algorithm are not influenced by either the poles’ location or the distribution of parallels and meridians, such problems will be theoretically solved. Inertial-frame (*i*-frame) and the Earth-fixed-frame (*e*-frame) navigation are able to satisfy this demand.

For example, using *e*-frame as the navigation frame, the differential equations of position, velocity and direction cosine matrix of attitude are [8,25]

(7) $$\begin{array}{l} {\dot{r}}^{e}=v^{e}\\ {\dot{v}}^{e}=C_{m}^{e}f^{m}-2〚\omega_{ie}^{e}〛v^{e}+g^{e}\\ {\dot{C}}_{m}^{e}=-〚\omega_{ie}^{e}〛C_{m}^{e}+C_{m}^{e}⟦\omega_{im}^{m}⟧ \end{array}\},$$

where ***r*** is the position vector, ***v*** is the velocity vector relative to the Earth, ***f*** is the specific force vector measured by the accelerometer unit, ***g*** is the gravity vector, $\omega_{ie}^{e}$ is the angular velocity vector of the *e*-frame relative to the *i*-frame, $\omega_{im}^{m}$ is the angular velocity vector of the measurement frame (*m*-frame) relative to the *i*-frame, the symbol 〚〛 indicates the formation of the antisymmetric matrix representation of the inner vector, and $C_{m}^{e}$ is the direction cosine matrix from *m*-frame to *e*-frame.

Based on Equation (7), the *e*-frame error model of the pure-inertial system is [8,25]

(8) $$\begin{array}{l} \delta {\dot{r}}^{e}=\delta v^{e}\\ \delta {\dot{v}}^{e}=〚f^{e}〛\Psi^{e}+C_{m}^{e}\Delta f^{m}-〚2\omega_{ie}^{e}〛\delta v^{e}+(\Gamma_{s}^{e}-〚\omega_{ie}^{e}〛〚\omega_{ie}^{e}〛)\delta r^{e}+C_{n}^{e}\delta g^{n}\\ {\dot{\Psi }}^{e}=-〚\omega_{ie}^{e}〛\Psi^{e}+\varepsilon^{e} \end{array}\},$$

where $\Gamma_{s}^{e}$ is the tensor of gravitational gradients and $\Psi^{e}$ is the psi angle in the *e*-frame. The main error sources of an INS are the accelerometer error, ${\Delta f}^{m}$, gyro drift rate, $\varepsilon^{e}$ and the gravity disturbance vector, ${\delta g}^{n}$. In the differential equations of navigation errors, the variables $C_{m}^{e}{\Delta f}^{m}$, $\varepsilon^{e}$, $C_{n}^{e}{\delta g}^{n}$, $f^{e}$ and $r^{e}$ change with the system’s position and then influence the equations’ solutions. If all of the error sources remain at the same level and the vehicle’s kinestate has no sudden change at different latitudes, solutions of Equation (8) will vary in the form of finite changes in the three components of a vector with a constant length as it points at different directions. Therefore, the *e*-frame algorithm could provide globally consistent navigation solutions without sharply increasing the number of errors in any region.

However, most applications prefer to use information about movements relative to the surface of the Earth, which the *e*-frame navigation outputs are not able to provide. This is the reason why almost all INSs use the *n*-frame as the navigation frame at low and mid latitudes. Although the *n*-frame becomes invalid near the poles, transverse and grid navigation based on alternative local-level frames have been presented and could ensure the same accuracy as traditional INSs in low or mid latitude regions. Yet, it should be noted that the transverse frame and the grid frame will become invalid near certain points on the equator. As a consequence, taking global navigation into consideration, a switch between traditional and polar navigation is required when the vehicle travels across the polar circles; this will be complicated and destroy the consistency of the entire navigation algorithm.

**Figure 1:**
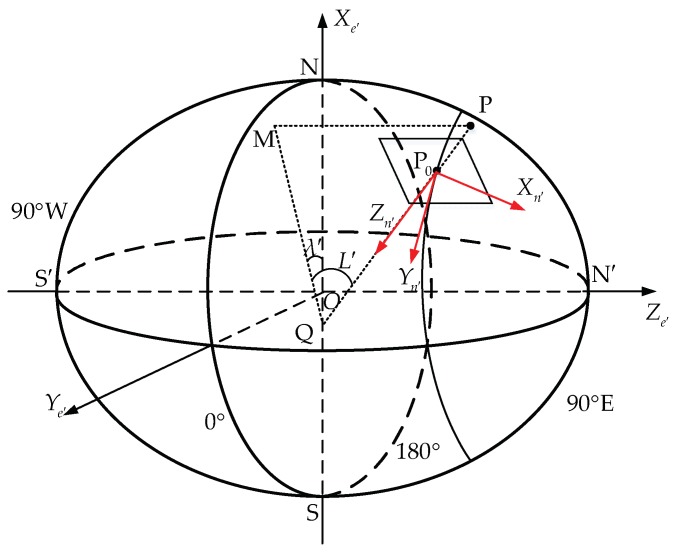
Definition of the modified transverse frame under the ellipsoidal Earth model.

Here, a hybrid navigation method is designed to combine the advantages of the *e*-frame system and the transverse system. The time integral in the INSs will be always carried out in the *e*-frame using Equation (7), namely, employing *e*-frame mechanization. However, outputs will be based on different frames according to the latitude. At low or mid latitudes, the system will output traditional *n*-frame positions, velocities and attitudes. In polar areas, the system will output navigation information based on the transverse frame. Thus, the switch is only needed in the output part and will be much easier to realize. The transverse frame used here is the modified one under the ellipsoidal Earth model [13] shown in Figure 1.

As shown in Figure 1, the transverse equatorial plane is the Greenwich meridian plane. The intersections of the 90° W and 90° E meridians with the equator are denoted as the transverse South Pole S′ and the transverse North Pole N′, respectively. $X_{e\prime }$, $Y_{e\prime }$ and $Z_{e\prime }$ denote the three axes of the transverse Earth-fixed frame (*e*′-frame). The $X_{e\prime }$ axis points toward the traditional North Pole N, the $Y_{e\prime }$ axis points toward the intersection of the equator and the Greenwich meridian, and the $Z_{e\prime }$ axis points toward N′. For a point, P, above the Earth’s surface, $P_{0}$ denotes the intersection of the local-level plane with its normal ${\mathrm{PP}}_{0}$. This normal will intersect with the $X_{e\prime }$ axis at point Q. Point M is the projection of P onto the transverse equatorial plane. The included angle of ${\mathrm{PP}}_{0}$ and the transverse equatorial plane is denoted as the transverse latitude, L′, the included angle of line QM and the $X_{e\prime }$ axis is denoted as the transverse longitude, λ′, and the transverse height, h′, has the same definition as the traditional one. $X_{n\prime }$, $Y_{n\prime }$ and $Z_{n\prime }$ denote the three axes of the transverse geographic frame (*n*′-frame). $X_{n\prime }$ points to the transverse north, which is along the intersecting line of plane PQM and the local-level plane. $Z_{n\prime }$ is the vector along the normal of the local-level plane and downwards. $Y_{n\prime }$ points to the transverse east to form the right-handed coordinate system.

The low and mid latitude part of the hybrid navigation algorithm and the corresponding error analysis were proposed in reference [25]. In this research, the high-latitude part, namely the hybrid transverse polar navigation method, will be mainly discussed. The calculations from the solutions of Equation (7) for the transverse position, velocity and attitude are given by the following equations.

(1) Transverse latitude and longitude

Based on their definitions and geometrical relationship, transverse latitude and longitude can be calculated iteratively using

(9) $$\begin{array}{l} L^{\prime }=\arctan\left(\frac{r_{y}^{e}}{\sqrt{{\left(r_{x}^{e}\right)}^{2}+{\left(r_{z}^{e}+e^{2}R_{N}\sin L\right)}^{2}}}\right)\\ \lambda^{\prime }=\arctan\left(\frac{r_{x}^{e}}{r_{z}^{e}+e^{2}R_{N}\sin L}\right)\\ h^{\prime }=\frac{\sqrt{{\left(r_{x}^{e}\right)}^{2}+{\left(r_{y}^{e}\right)}^{2}}}{\cos L}-R_{N} \end{array}\},$$

where [13]

(10) $$R_{N}=\frac{a}{{\left(1-e^{2}\cos^{2}L^{\prime }\cos^{2}\lambda^{\prime }\right)}^{1/2}}.$$

(2) Transverse velocity and attitude

The direction cosine matrix from the *e*-frame to the *n*′-frame could be calculated as

(11) $$C_{e}^{n^{\prime }}=\left(\begin{array}{ccc} -\sin\lambda^{\prime }\sin L^{\prime } & \cos L^{\prime } & -\cos\lambda^{\prime }\sin L^{\prime }\\ \cos\lambda^{\prime } & 0 & -\sin\lambda^{\prime }\\ -\sin\lambda^{\prime }\cos L^{\prime } & -\sin L^{\prime } & -\cos\lambda^{\prime }\cos L^{\prime } \end{array}\right).$$

Then, the transverse velocity relative to the Earth is

(12) $$v^{n^{\prime }}=C_{e}^{n\prime }v^{e}.$$

The direction cosine matrix from the body frame (*b*-frame) to the *n*′-frame is

(13) $$C_{b}^{n\prime }=C_{e}^{n\prime }C_{b}^{e},$$

The transverse attitude angles are defined as the transverse roll, φ′, the transverse pitch, θ′, and the transverse yaw, ψ′, which are the Euler angles calculated from $C_{b}^{n\prime }$.

## 4. Error Models of Pure-Inertial Hybrid Transverse Navigation

According to the analysis around Equation (8), error models in the *e*-frame are globally consistent. On the basis of the solutions of Equation (8), transverse navigation errors could be obtained by the following equations.

### 4.1. Transverse Position Errors

Transverse latitude and longitude errors can be calculated from the horizontal transverse position errors. According to reference [13] and the approximate relationship, $R_{N}\approx R_{M}\approx R$, they can be calculated with

(14) $$\begin{array}{l} \delta L^{\prime }=\frac{\delta r_{N\prime }}{R}\\ \delta \lambda^{\prime }=\frac{\delta r_{E\prime }}{R\cos L^{\prime }} \end{array}\},$$

where $R_{M}$ is the radius in the direction of the meridian, and *R* is the geocentric distance of the system. Using the transformation, ${\delta r}^{n\prime }=C_{e}^{n\prime }{\delta r}^{e}$, the vector consisting of transverse latitude and longitude errors $\delta \Lambda^{\prime }={\left(\delta L^{\prime }\delta \lambda^{\prime }\right)}^{T}$ can be calculated from the *e*-frame position errors, with

(15) $$\delta \Lambda^{\prime }=J_{3}^{\prime }\delta r^{e},$$

where

(16) $$J_{3}^{\prime }=\frac{1}{R}\left(\begin{array}{ccc} -\sin\lambda^{\prime }\sin L^{\prime } & \cos L^{\prime } & -\cos\lambda^{\prime }\sin L^{\prime }\\ \frac{\cos\lambda^{\prime }}{\cos L^{\prime }} & 0 & -\frac{\sin\lambda^{\prime }}{\cos L^{\prime }} \end{array}\right).$$

The height error can be calculated as

(17) $$\begin{array}{cc} \delta h^{\prime } & =-\delta r_{D^{\prime }}\\ & =\left(\begin{array}{ccc} \sin\lambda^{\prime }\cos L^{\prime } & \sin L^{\prime } & \cos\lambda^{\prime }\cos L^{\prime } \end{array}\right)\delta r^{e}\\ & =J_{2}^{\prime }{}^{T}\delta r^{e} \end{array}.$$

### 4.2. Transverse Velocity Error

Perturbing the equation, $v^{n\prime }{=C}_{e}^{n\prime }v^{e}$, yields the transverse velocity error

(18) $$\delta v^{n^{\prime }}=J_{1}^{\prime }\delta \Lambda^{\prime }+C_{e}^{n}\delta v^{e},$$

where

(19) $$J_{1}^{\prime }=\left(\begin{array}{cc} v_{D\prime } & -\sin L^{\prime }v_{E\prime }\\ 0 & \sin L^{\prime }v_{N\prime }+\cos L^{\prime }v_{D\prime }\\ -v_{N\prime } & -\cos L^{\prime }v_{E\prime } \end{array}\right).$$

### 4.3. Transverse Phi Angle and Attitude Errors

The phi angle, Φ, is the rotation vector that reflects the difference between the platform frame and the true frame. It is composed of the psi angle, Ψ, and δΘ [29,30]:

(20) Φ=Ψ+δΘ,

where δΘ represents the discrepancy between the computer frame and the true frame. When specified in the *n*′-frame, Equation (20) will be

(21) $$\Phi^{n^{\prime }}=-{A^{\prime }}_{3}\delta \Lambda \prime +C_{e}^{n^{\prime }}\Psi^{e},$$

where

(22) $${A^{\prime }}_{3}=\left(\begin{array}{cc} 0 & -\cos L^{\prime }\\ 1 & 0\\ 0 & \sin L^{\prime } \end{array}\right).$$

Then, the transverse attitude errors and the transverse phi angle have a relationship:

(23) $$\Phi^{n^{\prime }}=\left(\begin{array}{ccc} 0 & \sin\psi^{\prime } & -\cos\psi^{\prime }\cos\theta^{\prime }\\ 0 & -\cos\psi^{\prime } & -\sin\psi^{\prime }\cos\theta^{\prime }\\ -1 & 0 & \sin\theta^{\prime } \end{array}\right)\left(\begin{array}{c} \delta \psi^{\prime }\\ \delta \theta^{\prime }\\ \delta \phi^{\prime } \end{array}\right).$$

From comparing the formulae given in this section with those regarding the *n*-frame navigation errors [8,25], it can be seen that the methods to calculate the *n*′-frame and the *n*-frame navigation errors from the *e*-frame error models share the same structure, in spite of nuances in the calculation of some matrices. Therefore, with the *e*-frame error models being globally consistent, the global error models of the hybrid navigation scheme can achieve consistency in their forms.

## 5. Error Propagation Characteristics and Damping Technology

For high-precision INSs, damping is essential for long-term accuracy. To discuss the damping technology for hybrid transverse polar navigation, the first step is to analyze the pure-inertial error propagation characteristics.

No matter which frame is chosen for time integral, the error models will be the same when they have been transformed into one certain coordinate system. Therefore, the *n*′-frame error model shown in Equation (24) [13] can be used directly:

(24) $$\begin{array}{l} \delta {\dot{r}}^{n^{\prime }}=\delta v^{n^{\prime }}-〚\omega_{en^{\prime }}^{n^{\prime }}〛\delta r^{n^{\prime }}\\ \delta {\dot{v}}^{n^{\prime }}=-〚2\omega_{ie}^{n^{\prime }}+\omega_{en^{\prime }}^{n^{\prime }}〛\delta v^{n^{\prime }}+(\Gamma_{s}^{n^{\prime }}-〚\omega_{ie}^{n^{\prime }}〛〚\omega_{ie}^{n^{\prime }}〛)\delta r^{n^{\prime }}+〚f^{n^{\prime }}〛\Psi^{n^{\prime }}+\Delta f^{n^{\prime }}+\delta g^{n^{\prime }}\\ {\dot{\Psi }}^{n^{\prime }}=-〚\omega_{in^{\prime }}^{n^{\prime }}〛\Psi^{n^{\prime }}+\varepsilon^{n^{\prime }} \end{array}\}.$$

As the transverse height and the traditional height are actually the same, the characteristics of the vertical channel remain unchanged. Thus, the traditional vertical damping assisted by the altimeter is still an appropriate method for stabilizing the transverse vertical channel.

According to their definitions, in polar areas, the transverse latitude is quite small, and the transverse longitude is close to 0° (near the North Pole) or 180° (near the South Pole). Thus, $\omega_{ie}\cos\lambda^{\prime }$ is close to ${\pm \omega }_{ie}$, and the rates of transverse latitude and longitude are far less than $\omega_{ie}$ according to Equation (14), which means that the static and kinematic error models are approximately the same. Based on Equation (24), the state equation of the transverse horizontal channels is simplified as

(25) $$\left(\begin{array}{c} \delta {\dot{r}}_{N\prime }\\ \delta {\dot{r}}_{E\prime }\\ \delta {\dot{v}}_{N\prime }\\ \delta {\dot{v}}_{E\prime }\\ {\dot{\psi }}_{N\prime }\\ {\dot{\psi }}_{E\prime }\\ {\dot{\psi }}_{D\prime } \end{array}\right)=\left(\begin{array}{ccccccc} 0 & 0 & 1 & 0 & 0 & 0 & 0\\ 0 & 0 & 0 & 1 & 0 & 0 & 0\\ -\omega_{s}^{2} & 0 & 0 & -\omega_{1}^{\prime } & 0 & g & 0\\ 0 & -\omega_{s}^{2} & \omega_{1}^{\prime } & 0 & -g & 0 & 0\\ 0 & 0 & 0 & 0 & 0 & -\omega_{2}^{\prime } & \omega_{ie}\sin\lambda^{\prime }\\ 0 & 0 & 0 & 0 & \omega_{2}^{\prime } & 0 & -\omega_{3}^{\prime }\\ 0 & 0 & 0 & 0 & -\omega_{ie}\sin\lambda^{\prime } & \omega_{3}^{\prime } & 0 \end{array}\right)\left(\begin{array}{c} \delta r_{N\prime }\\ \delta r_{E\prime }\\ \delta v_{N\prime }\\ \delta v_{E\prime }\\ \psi_{N\prime }\\ \psi_{E\prime }\\ \psi_{D\prime } \end{array}\right)+\left(\begin{array}{c} 0\\ 0\\ \nabla_{N\prime }\\ \nabla_{E\prime }\\ \varepsilon_{N\prime }\\ \varepsilon_{E\prime }\\ \varepsilon_{D\prime } \end{array}\right),$$

where

(26) $$\begin{array}{l} \omega_{1}^{\prime }=2\omega_{ie}\cos\lambda^{\prime }\cos L^{\prime }\\ \omega_{2}^{\prime }=\omega_{ie}\cos\lambda^{\prime }\cos L^{\prime }\\ \omega_{3}^{\prime }=\omega_{ie}\cos\lambda^{\prime }\sin L^{\prime } \end{array}\}.$$

Using Equation (25), the characteristic equation is

(27) $$s\left(s^{2}+\omega_{ie}^{2}\right)\left[s^{4}+2\left(\omega_{s}^{2}+2\omega_{ie}^{2}\cos^{2}L^{\prime }\cos^{2}\lambda^{\prime }\right)s^{2}+\omega_{s}^{4}\right]=0.$$

The roots of Equation (27) consist of zero, a pair of purely imaginary numbers (${\pm j\omega }_{ie}$) and two pairs of complex numbers whose angular frequencies are approximately $\omega_{s}\pm \omega_{ie}\cos\;L^{\prime }\cos\;\lambda^{\prime }$. This result is similar to the conditions of traditional horizontal channels at low or mid latitudes. In the short term, Schuler oscillations dominate, and thus, the traditional horizontal damping network could be transplanted onto the *n*′-frame. The error curves of the damped system oscillate with a 24-h period.

**Figure 2:**
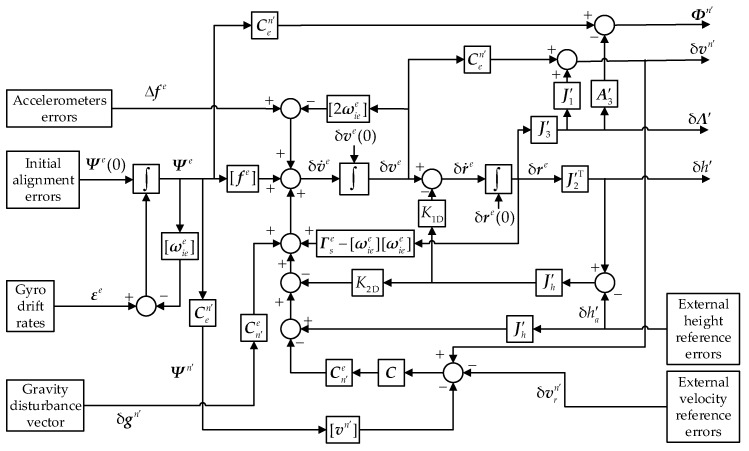
The error propagation of the damped hybrid transverse navigation.

Fortunately, as the mechanization is based on *e*-frame, the horizontal damping network has the same global structure. The damped *e*-frame differential equations are [8,25]

(28) $$\begin{array}{l} {\dot{r}}^{e}=v^{e}-K_{1D}J_{2}^{\prime }(h^{\prime }-h_{a}^{\prime })\\ {\dot{v}}^{e}=C_{m}^{e}f^{m}-〚2\omega_{ie}^{e}〛v^{e}+g^{e}-K_{2D}J_{2}^{\prime }(h^{\prime }-h_{a}^{\prime })-C_{n^{\prime }}^{e}C(v^{n^{\prime }}-v_{r}^{n^{\prime }})\\ {\dot{C}}_{m}^{e}=-〚\omega_{ie}^{e}〛C_{m}^{e}+C_{m}^{e}〚\omega_{im}^{m}〛 \end{array}\},$$

where $K_{1D}$ and $K_{2D}$ are the vertical damping coefficients, ***C*** represents the horizontal damping coefficient matrix, ${h\prime }_{a}$ is the external height reference and $v_{r}^{n\prime }$ is the external velocity reference. $C_{n\prime }^{e}C\left(v^{n\prime }-v_{r}^{n\prime }\right)$ is the only part that needs to be changed as the vehicle travels across the polar circles. At low and mid latitudes, it will be replaced by $C_{n}^{e}C\left(v^{n}-v_{r}^{n}\right)$. The error propagation of the damped hybrid transverse navigation is shown in Figure 2, and the coefficient matrices were given in Section 4.

## 6. Long-Term Error Analysis for Damped Hybrid Transverse Navigation

In order to improve the system’s performance, it is necessary to determine the contribution of each error source to the navigation errors. Although INSs are actually nonlinear and time-varying, it is possible to obtain the approximate analytical expressions of the navigation errors through proper simplification.

According to the error propagation of the damped hybrid transverse navigation shown in Figure 2, with the coupling between vertical and horizontal channels and terms related to $\omega_{ie}$, ${\dot{L}}^{\prime }$ and ${\dot{\lambda }}^{\prime }$ being neglected, transverse horizontal position and velocity errors satisfy the differential equations:

(29) $$\begin{array}{l} \delta {\dot{r}}_{N\prime }=\delta v_{N^{\prime }}\\ \delta {\dot{r}}_{E\prime }=\delta v_{E\prime }\\ \delta {\dot{v}}_{N\prime }+C\delta v_{N\prime }+\omega_{s}^{2}\delta r_{N\prime }=\Delta f_{N\prime }-f_{D\prime }\psi_{E\prime }+f_{E\prime }\psi_{D\prime }+C(-v_{D\prime }\psi_{E\prime }+v_{E\prime }\psi_{D\prime })\\ \delta {\dot{v}}_{E\prime }+C\delta v_{E\prime }+\omega_{s}^{2}\delta r_{E\prime }=\Delta f_{E\prime }+f_{D\prime }\psi_{N\prime }-f_{N\prime }\psi_{D\prime }+C(v_{D\prime }\psi_{N\prime }+v_{N\prime }\psi_{D\prime }) \end{array}\}.$$

Then, the Laplace transformation of horizontal position errors is

(30) $$\begin{array}{l} \delta r_{N\prime }\left(s\right)=\frac{\left(s+C\left(s\right)\right)\delta r_{N\prime 0}+\delta v_{N\prime 0}}{s^{2}+C\left(s\right)s+\omega_{s}^{2}}+\frac{C\left(s\right)\left(\psi_{D\prime }v_{E\prime }-\psi_{E\prime }v_{D\prime }\right)+\psi_{D\prime }f_{E\prime }-\psi_{E\prime }f_{D\prime }}{s^{2}+C\left(s\right)s+\omega_{s}^{2}}\\ \delta r_{E\prime }\left(s\right)=\frac{\left(s+C\left(s\right)\right)\delta r_{E\prime 0}+\delta v_{E\prime 0}}{s^{2}+C\left(s\right)s+\omega_{s}^{2}}+\frac{C\left(s\right)\left(-\psi_{D\prime }v_{N\prime }+\psi_{N\prime }v_{D\prime }\right)-\psi_{D\prime }f_{N\prime }+\psi_{E\prime }f_{D\prime }}{s^{2}+C\left(s\right)s+\omega_{s}^{2}} \end{array}\}.$$

Based on the final-value theorem and Equation (21), the steady-state transverse horizontal position, velocity and attitude errors are:

(31) $$\begin{array}{l} \delta L^{\prime }=\psi_{E\prime }+\frac{\Delta f_{N\prime }}{g}\\ \delta \lambda^{\prime }\cos L^{\prime }=-\psi_{N\prime }+\frac{\Delta f_{E\prime }}{g}\\ \delta v_{N\prime }=R\delta {\dot{L}}^{\prime }=R\varepsilon_{E\prime }+\frac{\Delta {\dot{f}}_{N\prime }}{\omega_{s}^{2}}\\ \delta v_{E\prime }=R\delta {\dot{\lambda }}^{\prime }\cos L^{\prime }=-R\varepsilon_{N\prime }+\frac{\Delta {\dot{f}}_{E\prime }}{\omega_{s}^{2}}\\ \phi_{N\prime }=\frac{\Delta f_{E\prime }}{g}\\ \phi_{E\prime }=-\frac{\Delta f_{N\prime }}{g}\\ \phi_{D\prime }=\psi_{D\prime }+(\psi_{N\prime }-\frac{\Delta f_{E\prime }}{g})\tan L^{\prime } \end{array}\}.$$

If the right side is calculated from the *e*-frame values, Equation (31) can be written as

(32) $$\begin{array}{l} \delta L^{\prime }=\psi_{x}^{e}\cos\lambda^{\prime }-\psi_{z}^{e}\sin\lambda^{\prime }+\frac{1}{g}(-\Delta f_{x}^{e}\sin\lambda^{\prime }\sin L^{\prime }+\Delta f_{y}^{e}\cos L^{\prime }-\Delta f_{z}^{e}\cos\lambda^{\prime }\sin L^{\prime })\\ \delta \lambda^{\prime }\cos L^{\prime }=\psi_{x}^{e}\sin\lambda^{\prime }\sin L^{\prime }-\psi_{y}^{e}\cos L^{\prime }+\psi_{z}^{e}\cos\lambda^{\prime }\sin L^{\prime }+\frac{1}{g}(\Delta f_{x}^{e}\cos\lambda^{\prime }-\Delta f_{z}^{e}\sin\lambda^{\prime })\\ \delta v_{N\prime }=R[{\dot{\psi }}_{x}^{e}\cos\lambda^{\prime }-{\dot{\psi }}_{z}^{e}\sin\lambda^{\prime }+\frac{1}{g}(-\Delta {\dot{f}}_{x}^{e}\sin\lambda^{\prime }\sin L^{\prime }+\Delta {\dot{f}}_{y}^{e}\cos L^{\prime }-\Delta {\dot{f}}_{z}^{e}\cos\lambda^{\prime }\sin L^{\prime })]\\ \delta v_{E\prime }=R[{\dot{\psi }}_{x}^{e}\sin\lambda^{\prime }\sin L^{\prime }-{\dot{\psi }}_{y}^{e}\cos L^{\prime }+{\dot{\psi }}_{z}^{e}\cos\lambda^{\prime }\sin L^{\prime }+\frac{1}{g}(\Delta {\dot{f}}_{x}^{e}\cos\lambda^{\prime }-\Delta {\dot{f}}_{z}^{e}\sin\lambda^{\prime })]\\ \phi_{N\prime }=\frac{1}{g}(\Delta f_{x}^{e}\cos\lambda^{\prime }-\Delta f_{z}^{e}\sin\lambda^{\prime })\\ \phi_{E\prime }=-\frac{1}{g}(-\Delta f_{x}^{e}\sin\lambda^{\prime }\sin L^{\prime }+\Delta f_{y}^{e}\cos L^{\prime }-\Delta f_{z}^{e}\cos\lambda^{\prime }\sin L^{\prime })\\ \phi_{D\prime }=-\frac{1}{\cos L^{\prime }}(\sin\lambda^{\prime }\psi_{x}^{e}+\cos\lambda^{\prime }\psi_{z}^{e})-\frac{\tan L^{\prime }}{g}(\Delta f_{x}^{e}\cos\lambda^{\prime }-\Delta f_{z}^{e}\sin\lambda^{\prime }) \end{array}\}.$$

Equation (32) uses the *e*-frame accelerometer errors and gyro drifts, which are related to the orientation of the *m*-frame and the error models of inertial measurement units (IMUs). As the mechanization of space-stable INSs best matches the hybrid transverse algorithm, the detailed analytical expressions of long-term navigation errors are given for such systems.

### 6.1. Error Model of Accelerometer Unit

For low-speed cruising, the specific forces measured can be approximately expressed as

(33) $$\left(\begin{array}{c} f_{x}^{P}\\ f_{y}^{P}\\ f_{z}^{P} \end{array}\right)\approx C_{n^{\prime }}^{P}\left(\begin{array}{c} 0\\ 0\\ -g \end{array}\right)=g\left(\begin{array}{c} A_{\phi }\cos\left(\gamma_{2}^{P}+\phi \right)\\ A_{\phi }\sin\left(\gamma_{2}^{P}+\phi \right)\\ \cos\lambda^{\prime }\cos L^{\prime } \end{array}\right),$$

where

(34) $$\begin{array}{l} A_{\phi }=\sqrt{1-\cos^{2}L^{\prime }\cos^{2}\lambda^{\prime }}\\ \sin\phi =\sin L^{\prime }/A_{\phi }\\ \cos\phi =\cos L^{\prime }\sin\lambda^{\prime }/A_{\phi } \end{array}\}.$$

The superscript *P* denotes the platform frame (*P*-frame), and $\gamma_{2}^{P}$ represents the rotation of the space-stable platform, which is $\omega_{ie}t$.

In the *P*-frame, the error model of the three-axis accelerometer unit is [28]

(35) $$\left(\begin{array}{c} \Delta f_{x}^{P}\\ \Delta f_{y}^{P}\\ \Delta f_{z}^{P} \end{array}\right)=\left(\begin{array}{ccc} \frac{\Delta SF_{x}}{SF_{x}} & -\Delta \beta_{xz} & \Delta \beta_{xy}\\ \Delta \beta_{yz} & \frac{\Delta SF_{y}}{SF_{y}} & -\Delta \beta_{yx}\\ -\Delta \beta_{zy} & \Delta \beta_{zx} & \frac{\Delta SF_{z}}{SF_{z}} \end{array}\right)\left(\begin{array}{c} f_{x}^{P}\\ f_{y}^{P}\\ f_{z}^{P} \end{array}\right)-\left(\begin{array}{c} \nabla_{x}\\ \nabla_{y}\\ \nabla_{z} \end{array}\right),$$

where ∇, $\frac{\Delta SF}{SF}$ and Δβ represent the zero bias, scale factor error and installation angle error with corresponding subscripts denoting the direction. Combining Equation (35) with Equation (33) and transforming the result to *e*-frame yields

(36) $$\begin{array}{cc} \frac{\Delta f_{x}^{e}}{g}= & -\left(\frac{\nabla_{x}}{g}-\Delta \beta_{xy}\cos L^{\prime }\cos\lambda^{\prime }\right)\cos\gamma_{2}^{P}-\left(\frac{\nabla_{y}}{g}+\Delta \beta_{yx}\cos L^{\prime }\cos\lambda^{\prime }\right)\sin\gamma_{2}^{P}\\ & +A_{\phi }\left(\frac{\Delta \beta_{yz}}{2}-\frac{\Delta \beta_{xz}}{2}\right)\sin\left(2\gamma_{2}^{P}+\phi \right)+A_{\phi }\left(\frac{\Delta SF_{x}}{2SF_{x}}-\frac{\Delta SF_{y}}{2SF_{y}}\right)\cos\left(2\gamma_{2}^{P}+\phi \right)\\ & -A_{\phi }\left(\frac{\Delta \beta_{yz}}{2}+\frac{\Delta \beta_{xz}}{2}\right)\sin\phi +A_{\phi }\left(\frac{\Delta SF_{x}}{2SF_{x}}+\frac{\Delta SF_{y}}{2SF_{y}}\right)\cos\phi \\ \frac{\Delta f_{y}^{e}}{g}= & \left(\frac{\nabla_{x}}{g}-\Delta \beta_{xy}\cos L^{\prime }\cos\lambda^{\prime }\right)\sin\gamma_{2}^{P}-\left(\frac{\nabla_{y}}{g}+\Delta \beta_{yx}\cos L^{\prime }\cos\lambda^{\prime }\right)\cos\gamma_{2}^{P}\\ & +A_{\phi }\left(\frac{\Delta \beta_{yz}}{2}-\frac{\Delta \beta_{xz}}{2}\right)\cos\left(2\gamma_{2}^{P}+\phi \right)-A_{\phi }\left(\frac{\Delta SF_{x}}{2SF_{x}}-\frac{\Delta SF_{y}}{2SF_{y}}\right)\sin\left(2\gamma_{2}^{P}+\phi \right)\\ & +A_{\phi }\left(\frac{\Delta \beta_{yz}}{2}+\frac{\Delta \beta_{xz}}{2}\right)\cos\phi +A_{\phi }\left(\frac{\Delta SF_{x}}{2SF_{x}}+\frac{\Delta SF_{y}}{2SF_{y}}\right)\sin\phi \\ \frac{\Delta f_{z}^{e}}{g}= & -\frac{\nabla_{z}}{g}+\frac{\Delta SF_{z}}{SF_{z}}\cos L^{\prime }\cos\lambda^{\prime }+\Delta \beta_{zx}A_{\phi }\sin\left(\gamma_{2}^{P}+\phi \right)-\Delta \beta_{zy}A_{\phi }\cos\left(\gamma_{2}^{P}+\phi \right) \end{array}\}.$$

### 6.2. Gyro Dift and Psi Angle

Based on the fundamental gyro drift models [25,28,31], the residual drift rates of gyros on the space-stable platform after proper calibration and error compensation can be modeled as

(37) $$\begin{array}{l} \Delta \omega_{1x}=\Delta \varepsilon_{1x}+A_{\phi }\Delta {\bar{d}}_{11}\cos\left(\gamma_{2}^{P}+\phi \right)\\ \Delta \omega_{1y}=\Delta \varepsilon_{1y}+A_{\phi }\Delta {\bar{d}}_{11}\sin\left(\gamma_{2}^{P}+\phi \right) \end{array}\}\;\mathrm{polar}\mathrm{gyro},$$

(38) $$\Delta \omega_{2z}=\Delta {\bar{\varepsilon }}_{2z}+\Delta d_{22}\cos L^{\prime }\cos\lambda^{\prime }A_{\phi }\cos\left(\gamma_{2}^{P}+\phi \right)\;\mathrm{equatorial}\mathrm{gyro},$$

where $\Delta \varepsilon_{1x}$, $\Delta \varepsilon_{1y}$ and $\Delta {\bar{\varepsilon }}_{2z}$ are called the $g^{0}$ coefficients, and $\Delta {\bar{d}}_{11}$ and $\Delta d_{22}$ are called the $g^{1}$ coefficients.

Substituting Equations (37) and (38) into the differential equations of the perturbed motion of the space-stable platform [25] yields the three components of the psi angle as

(39) $$\begin{array}{cc} \psi_{x}^{e}\left(t\right) & \approx \frac{\Delta {\bar{d}}_{11}A_{\phi }}{\omega_{ie}}\left(\sin\phi +\sin\left(\omega_{ie}t-\phi_{0}\right)\right)+\psi_{x}^{e}\left(0\right)\cos\omega_{ie}t\\ & +\psi_{y}^{e}\left(0\right)\sin\omega_{ie}t+\Delta \varepsilon_{1x}t\cos\left[\omega_{ie}t+\gamma_{2}^{P}\left(0\right)\right]+\Delta \varepsilon_{1y}t\sin\left[\omega_{ie}t+\gamma_{2}^{P}\left(0\right)\right]\\ \psi_{y}^{e}\left(t\right) & \approx -\frac{\Delta {\bar{d}}_{11}A_{\phi }}{\omega_{ie}}\left(\cos\phi -\cos\left(\omega_{ie}t-\phi_{0}\right)\right)-\psi_{x}^{e}\left(0\right)\sin\omega_{ie}t\\ & +\psi_{y}^{e}\left(0\right)\cos\omega_{ie}t-\Delta \varepsilon_{1x}t\sin\left[\omega_{ie}t+\gamma_{2}^{P}\left(0\right)\right]+\Delta \varepsilon_{1y}t\cos\left[\omega_{ie}t+\gamma_{2}^{P}\left(0\right)\right]\\ \psi_{z}^{e}\left(t\right) & =\psi_{z}^{e}\left(0\right)+\Delta {\bar{\varepsilon }}_{2z}t+\frac{\Delta d_{22}\cos L\prime \cos l\prime A_{\phi }}{\omega_{ie}+\dot{\phi }}\left\{\sin\left[\omega_{ie}t+\gamma_{2}^{P}\left(0\right)+\phi \right]-\sin\left[\gamma_{2}^{P}\left(0\right)+\phi_{0}\right]\right\} \end{array}\},$$

where the time-varying characteristic of ϕ in polar areas has been considered, and $\phi_{0}$ is its initial value.

### 6.3. Long-Term Contribution of Main Error Sources

Substituting Equations (36) and (39) into Equation (32) yields the approximate analytical expressions of the long-term navigation errors of the low-speed space-stable INSs, which reflect the propagation of the main error sources. As the horizontal position errors and the azimuth error are the most concerning, the impacts of different error coefficients on them are listed in Table 1. Second-order small quantities have been neglected based on the fact that the sine and tangent of transverse latitude and longitude gradually approach zero as the vehicle moves towards the poles.

From Table 1, it can be concluded that:

(1) The terms dominated in transverse latitude and longitude errors are the slowly divergent 24-h period oscillation caused by $\Delta \varepsilon_{1x}$ and $\Delta \varepsilon_{1y}$ and the persistent 24-h period oscillation caused by $\nabla_{x}$, $\nabla_{y}$, $\Delta \beta_{xy}$, $\Delta \beta_{yx}$, $\Psi_{x}^{e}(0)$ and $\Psi_{y}^{e}(0)$.
(2) For the transverse azimuth error, the linear growth caused by $\Delta {\bar{\varepsilon }}_{2z}$ predominates. The initial alignment error, $\Psi_{z}^{e}(0),$ will induce a constant bias. The slowly divergent 24-h period oscillation caused by $\Delta \varepsilon_{1x}$ and $\Delta \varepsilon_{1y}$ also exists. Although it is attenuated by $\sin\lambda^{\prime }$, it is possible for this fluctuation to become apparent after a sufficient length of time.
(3) All the dominant terms in error curves will account for larger proportions, and others gradually disappear as the system comes close to the poles. Specifically, transverse latitude and longitude errors will symmetrically and divergently oscillate, and the transverse azimuth error curve will be an oblique line when the system is close enough to the poles.

where

(40) $$\begin{array}{l} A_{\vartheta }=\sqrt{1-\sin^{2}L^{\prime }\cos^{2}\lambda^{\prime }}\\ \sin\vartheta =\sin L^{\prime }\sin\lambda^{\prime }/A_{\vartheta }\\ \cos\vartheta =\cos L^{\prime }/A_{\vartheta } \end{array}\}$$

## 7. Simulation and Discussion

Simulation results of the proposed method are presented in this section. Outputs of gyros and accelerometers were generated by the space-stable INS simulator. The errors of IMUs were generated based on the error models presented in Section 6, where the $g^{0}$ and $g^{1}$ coefficients of gyro drifts were all $10^{-4}\;{}^{\circ}/h$, the initial alignment errors were 10″, and the zero biases, scale factor errors and installation errors of the accelerometers were ${2\times 10}^{-5}$ *g*, 10 ppm and 10″, respectively. These values of error parameters were chosen based on the general performance of IMUs in the navigation grade (1 nm/h) class [31,32] and a certain high-precision INS [33]. In addition, it is assumed that proper calibration and error compensation have been carried out during the initialization.

### 7.1. e-Frame Navigation Errors at Different Latitudes

**Figure 3:**
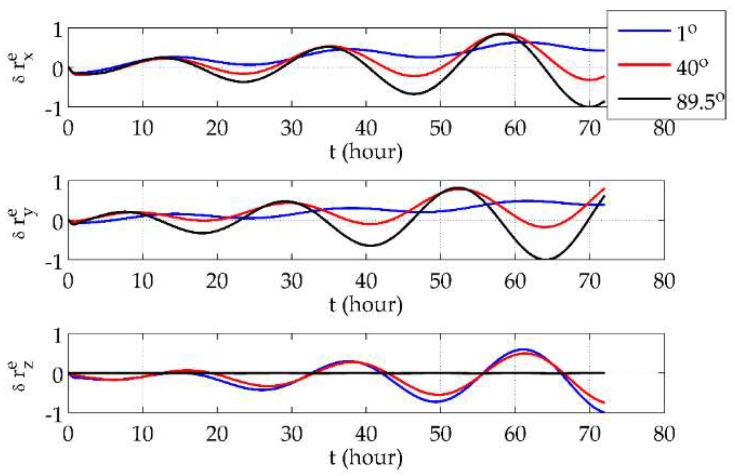
The *e*-frame position errors at different latitudes.

**Figure 4:**
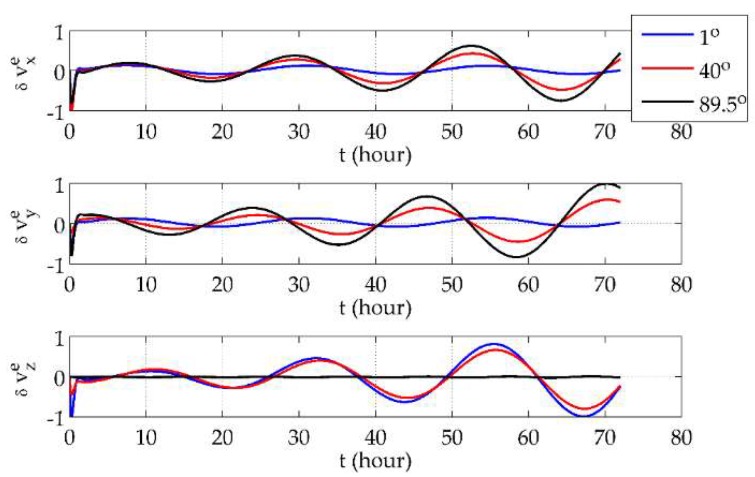
The *e*-frame velocity errors at different latitudes.

**Figure 5:**
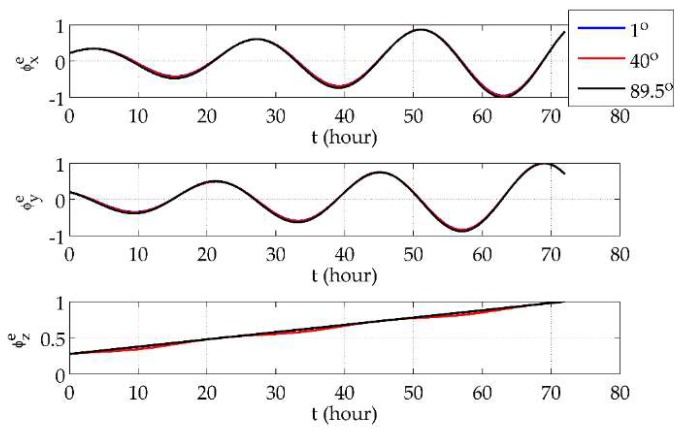
The *e*-frame phi angles at different latitudes.

First, it was demonstrated that the *e*-frame mechanization would not be affected by the system’s position. The vehicle was set to move along the parallels of 1° N, 40° N and 89.5° N eastwards at a speed of 5 m/s for 3 days. The *e*-frame position errors, velocity errors and phi angles are shown in Figure 3, Figure 4 and Figure 5, respectively, where all of the error curves are normalized by the maximum absolute values in the corresponding directions among the three latitudes.

From Figure 3 and Figure 4, it can be seen that position and velocity errors at different latitudes showed no sharp increases but only variance in the distribution of the components in three directions. At low latitudes, components in the *x* and *y* directions were distinctly smaller than the one in the *z* direction, while the situation at high latitudes is the opposite. Also, the drifts in the *x*- and *y*-direction position errors were more obvious at lower latitudes. As for the *e*-frame phi angles, Figure 5 shows that they were finite and almost unrelated to the latitude. Such results validate the use of the *e*-frame mechanization globally.

### 7.2. Long-Term Hybrid Transverse Navigation Errors in Polar Areas

**Figure 6:**
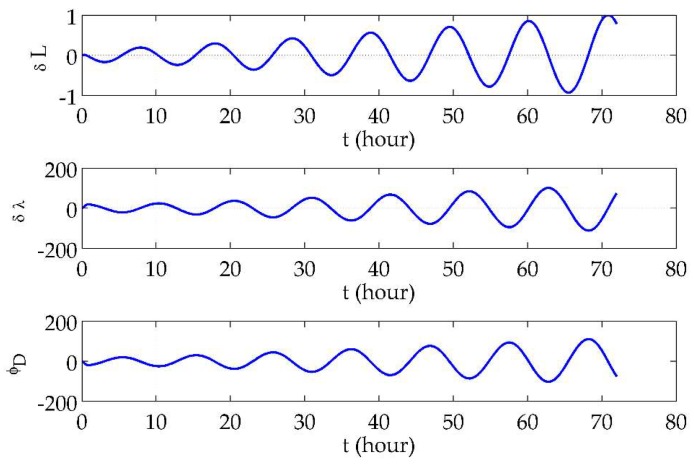
Horizontal position errors and the azimuth error using the traditional algorithm.

**Figure 7:**
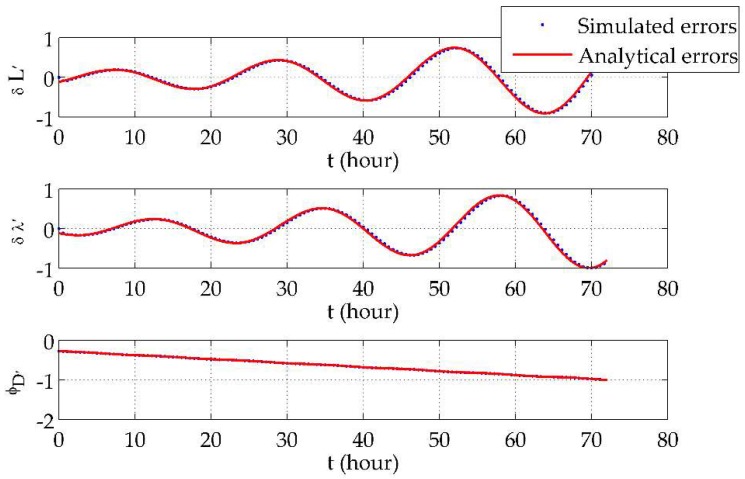
Horizontal position errors and the azimuth error using the hybrid transverse algorithm, and comparisons with the analytical expressions.

The initial position was set to be ${\left(89.5{}^{\circ}\;N\;116{}^{\circ}\;E\;0\;m\right)}^{T}$, which is quite an extreme condition. The simulation involved the vehicle moving along the 89.5° N parallel at a speed of 5 m/s eastwards for 3 days. The horizontal position errors and the azimuth error using the traditional navigation algorithm and the hybrid transverse navigation algorithm are shown in Figure 6 and Figure 7, respectively, where all error curves are normalized by the maximum absolute values of the traditional latitude errors.

From Figure 6, it can be seen that the longitude error and azimuth error were far larger than the latitude error and that they became over 100 times larger on the third day. Moreover, oscillations in the error curves had about seven cycles within 3 days, which is obviously different from the long-term oscillation characteristics at low and mid latitudes. The angular frequency of the oscillations actually matches ($\dot{\lambda }$ + *ω_ie_*) and conforms to the proposal that in polar areas, movements along the parallels will result in more drastic fluctuations in navigation errors.

The error curves shown in Figure 7 do not have similar problems to Figure 6. Using the hybrid transverse navigation algorithm, navigation errors are at normal levels and their oscillation period is approximately 24 h. Such results are consistent with use of the traditional algorithm at low and mid latitudes. In addition, analytical errors were calculated based on Table 1 and compared with the simulated errors in Figure 7. The results show that they fit with each other and validate the analytical expressions in Section 6. At 89.5° N, the transverse latitude errors and longitude errors were mainly due to symmetrical divergent oscillation caused by the *x*- and *y*-direction $g^{0}$ gyro drift coefficients, and the transverse azimuth error was mainly due to the linear drift caused by the *z*-direction $g^{0}$ gyro drift coefficient. The analytical expressions theoretically explain the error curves shown in existing research and could be used to assist the design of high-precision INSs for polar applications.

## 8. Conclusions

This research proposed a hybrid transverse polar navigation method based on a combination of *e*-frame navigation and transverse navigation. A kinematic pure-inertial error analysis was conducted to introduce proper damping technology for high-precision and long-term INSs in polar areas. According to the theoretical analysis, the error models proposed can retain the same structure globally and the damping network can be globally consistent. This means that the hybrid global navigation is mathematically convenient and can be easily implemented by merely switching the coordinate system for navigation outputs, without any break in the core integral process. The simulation results showed that the *e*-frame mechanization is not influenced by the system’s position, and that the proposed polar navigation method can ensure the same accuracy and oscillation characteristics as the traditional algorithm in low and mid latitude regions. The proposed polar navigation method could be used in INSs of different types, and it is most suitable for the space-stable INSs, in principle. The analytical expressions of the long-term navigation errors in the damped space-stable INSs were deduced to figure out the contributions of main error sources, which were also validated by the simulation results. They could help to further improve the systems’ polar accuracy.

## Figures and Tables

**Table 1:** Long-term horizontal position errors and azimuth error induced by error coefficients in the space-stable inertial navigation systems (INSs).

Error Coefficients		Corresponding Navigation Errors		
		$\delta L^{\prime }$	$\delta \lambda^{\prime }\cos\;L^{\prime }$	$\phi_{D^{\prime }}$
Gyro drift model coefficients	$\Delta \varepsilon_{1x}$	$\Delta \varepsilon_{1x}{t\cos\;\lambda \prime \cos\omega }_{ie}t$	$A_{\vartheta }{\Delta \varepsilon }_{1x}t\sin\left(\omega_{ie}t+\vartheta \right)$	$-\frac{1}{\cos L\prime }{\Delta \varepsilon }_{1x}{t\sin\;\lambda^{\prime }\cos\omega }_{ie}t$
	$\Delta \varepsilon_{1y}$	$\Delta \varepsilon_{1y}{t\cos\;\lambda^{\prime }\sin\omega }_{ie}t$	$A_{\vartheta }{\Delta \varepsilon }_{1y}t\cos\left(\omega_{ie}t+\vartheta \right)$	$-\frac{1}{\cos L\prime }{\Delta \varepsilon }_{1y}{t\sin\;\lambda^{\prime }\sin\omega }_{ie}t$
	$\Delta {\bar{\varepsilon }}_{2z}$	$-\Delta {\bar{\varepsilon }}_{2z}t\sin\;\lambda^{\prime }$	$\Delta {\bar{\varepsilon }}_{2z}t\cos\;\lambda^{\prime }\sin L^{\prime }$	$-\frac{1}{\cos L\prime }\Delta {\bar{\varepsilon }}_{2z}t\cos\;\lambda^{\prime }$
	$\Delta {\bar{d}}_{11}$	$\frac{\Delta {\bar{d}}_{11}A_{\phi }\cos\lambda \prime }{\omega_{ie}}\left[\begin{array}{c} \sin\phi \\ +\sin\left(\omega_{ie}t-\phi_{0}\right) \end{array}\right]$	$\frac{\Delta {\bar{d}}_{11}A_{\phi }A_{\vartheta }}{\omega_{ie}}\left[\begin{array}{c} \cos(\vartheta -\phi )\\ -\cos\left(\omega_{ie}t+\vartheta -\phi_{0}\right) \end{array}\right]$	≈0
	$\Delta d_{22}$	≈0	≈0	$-\frac{\Delta d_{22}A_{\phi }\cos^{2}\lambda^{\prime }}{\omega_{ie}+\dot{\phi }}\left[\begin{array}{c} \sin(\omega_{ie}t+\phi )\\ -\sin\phi_{0} \end{array}\right]$
Accelerometer error model coefficients	$\frac{\Delta {SF}_{x}}{{SF}_{x}}$	$\frac{\Delta {SF}_{x}}{2{SF}_{x}}\left[\begin{array}{c} \cos^{2}\lambda \prime \cos L\prime \sin L\prime \\ -A_{\phi }A_{\vartheta }\sin\left({2\omega }_{ie}t+\vartheta +\phi \right) \end{array}\right]$	$\frac{\Delta {SF}_{x}}{2{SF}_{x}}\left[\begin{array}{c} A_{\phi }\cos\;\lambda^{\prime }\cos\left({2\omega }_{ie}t+\phi \right)\\ +\cos L\prime \cos\;\lambda^{\prime }\sin\;\lambda^{\prime } \end{array}\right]$	≈0
	$\frac{\Delta {SF}_{y}}{{SF}_{y}}$	$\frac{\Delta {SF}_{y}}{2{SF}_{y}}\left[\begin{array}{c} \cos^{2}\lambda^{\prime }\cos L^{\prime }\sin L^{\prime }\\ {+A}_{\phi }A_{\vartheta }\sin\left({2\omega }_{ie}t+\vartheta +\phi \right) \end{array}\right]$	$\frac{\Delta {SF}_{y}}{2{SF}_{y}}\left[\begin{array}{c} -A_{\phi }\cos\;\lambda^{\prime }\cos\left({2\omega }_{ie}t+\phi \right)\\ +\cos L^{\prime }\cos\;\lambda^{\prime }\sin\;\lambda^{\prime } \end{array}\right]$	≈0
	$\frac{\Delta {SF}_{z}}{{SF}_{z}}$	$-\frac{\Delta {SF}_{z}}{{SF}_{z}}\cos^{2}\lambda^{\prime }\cos L^{\prime }\sin L^{\prime }$	$-\frac{\Delta {SF}_{z}}{{SF}_{z}}\cos L^{\prime }\sin\lambda^{\prime }\cos\lambda^{\prime }$	≈0
	$\frac{\nabla_{x}}{g}$	$\frac{\nabla_{x}}{g}A_{\vartheta }\sin\left(\omega_{ie}t+\vartheta \right)$	$-\frac{\nabla_{x}}{g}{\cos\;\lambda^{\prime }\cos\omega }_{ie}t$	$\frac{\nabla_{x}}{g}{\cos\;\lambda^{\prime }\tan L\prime \cos\omega }_{ie}t$
	$\frac{\nabla_{y}}{g}$	$-\frac{\nabla_{y}}{g}A_{\vartheta }\cos\left(\omega_{ie}t+\vartheta \right)$	$-\frac{\nabla_{y}}{g}{\cos\;\lambda^{\prime }\sin\omega }_{ie}t$	$\frac{\nabla_{y}}{g}{\cos\;\lambda^{\prime }\tan L^{\prime }\sin\omega }_{ie}t$
	$\frac{\nabla_{z}}{g}$	$\frac{\nabla_{z}}{g}\cos\;\lambda^{\prime }\sin L\prime$	$\frac{\nabla_{z}}{g}\sin\;\lambda^{\prime }$	≈0
	$\Delta \beta_{xy}$	$-\Delta \beta_{xy}{\cos L^{\prime }\cos\;\lambda^{\prime }A}_{\vartheta }\sin\left(\omega_{ie}t+\vartheta \right)$	$\Delta \beta_{xy}{\cos L\prime \cos}^{2}\;{\lambda^{\prime }\cos\omega }_{ie}t$	$-\Delta \beta_{xy}{\sin L\prime \cos}^{2}\;{\lambda \prime \cos\omega }_{ie}t$
	$\Delta \beta_{yx}$	$-\Delta \beta_{yx}{\cos L\prime \cos\;\lambda^{\prime }A}_{\vartheta }\cos\left(\omega_{ie}t+\vartheta \right)$	$-\Delta \beta_{yx}{\cos L\prime \cos}^{2}\;{\lambda^{\prime }\sin\omega }_{ie}t$	$\Delta \beta_{yx}{\sin L\prime \cos}^{2}\;{\lambda^{\prime }\sin\omega }_{ie}t$
	$\Delta \beta_{xz}$	$\frac{\Delta \beta_{xz}}{2}\left(\begin{array}{c} \sin\;\lambda^{\prime }-\\ A_{\phi }A_{\vartheta }\cos\left({2\omega }_{ie}t+\vartheta +\phi \right) \end{array}\right)$	$-\frac{\Delta \beta_{xz}}{2}\left(\begin{array}{c} A_{\phi }\cos\;\lambda^{\prime }\sin\left({2\omega }_{ie}\;t+\phi \right)\\ +\sin L^{\prime }\cos\;\lambda^{\prime } \end{array}\right)$	≈0
	$\Delta \beta_{zx}$	≈0	≈0	≈0
	$\Delta \beta_{yz}$	$\frac{\Delta \beta_{yz}}{2}\left(\begin{array}{c} \sin\;\lambda^{\prime }+\\ A_{\phi }A_{\vartheta }\cos\left({2\omega }_{ie}t+\vartheta +\phi \right) \end{array}\right)$	$\frac{\Delta \beta_{yz}}{2}\left(\begin{array}{c} A_{\phi }\cos\;\lambda^{\prime }\sin\left({2\omega }_{ie}t+\phi \right)\\ -\sin L^{\prime }\cos\;\lambda^{\prime } \end{array}\right)$	≈0
	$\Delta \beta_{zy}$	≈0	≈0	≈0
Initial alignment errors	$\Psi_{x}^{e}\left(0\right)$	$\Psi_{x}^{e}\left(0\right){\;\cos\;\lambda^{\prime }\cos\omega }_{ie}t$	$\Psi_{x}^{e}\left(0\right)A_{\vartheta }\sin\left(\omega_{ie}t+\vartheta \right)$	$-\frac{1}{\cos L^{\prime }}\Psi_{x}^{e}\left(0\right){\sin\;\lambda^{\prime }\cos\omega }_{ie}t$
	$\Psi_{y}^{e}\left(0\right)$	$\Psi_{y}^{e}\left(0\right)\;{\cos\;\lambda^{\prime }\sin\omega }_{ie}t$	$-\Psi_{y}^{e}\left(0\right)A_{\vartheta }\cos\left(\omega_{ie}t+\vartheta \right)$	$-\frac{1}{\cos L\prime }\Psi_{y}^{e}\left(0\right){\sin\;\lambda^{\prime }\sin\omega }_{ie}t$
	$\Psi_{z}^{e}\left(0\right)$	$-\Psi_{z}^{e}\left(0\right)\sin\;\lambda^{\prime }$	$\Psi_{z}^{e}\left(0\right)\cos\;\lambda^{\prime }\sin L^{\prime }$	$-\frac{1}{\cos L^{\prime }}\Psi_{z}^{e}\left(0\right)\;\cos\;\lambda^{\prime }$
